# Understanding chronic non-communicable diseases in Latin America: towards an equity-based research agenda

**DOI:** 10.1186/1744-8603-7-36

**Published:** 2011-10-07

**Authors:** Fernando G De Maio

**Affiliations:** 1 Department of Sociology, DePaul University, 990 W. Fullerton Ave., Suite 1100, Chicago, IL 60614, USA

**Keywords:** Latin America, chronic disease, risk factors, social justice

## Abstract

Although chronic non-communicable diseases are traditionally depicted as diseases of affluence, growing evidence suggests they strike along the fault lines of social inequality. The challenge of understanding how these conditions shape patterns of population health in Latin America requires an inter-disciplinary lens. This paper reviews the burden of chronic non-communicable diseases in the region and examines key myths surrounding their prevalence and distribution. It argues that a social justice approach rooted in the idea of health inequity needs to be at the core of research in this area, and concludes with discussion of a new approach to guide empirical research, the 'average/deprivation/inequality' framework.

## Introduction

A population's health is a critical indicator of the quality of its social fabric. For this reason dismay is generated by the well-known statistics: while men in many parts of the industrialised world may expect to live, on average, to see their late seventies and women in many countries may expect to live well into their eighties, billions of people around the world live in dramatically different 'epidemiological worlds' [1,2]. These worlds are characterized by substantially worse aggregate indicators, including life expectancies in the low forties. Different 'epidemiological worlds' are characterized by high levels of social inequity in terms of socioeconomic resources, health status, and by higher rates of exposure to a wide range of health risks. These risks are varied - from the structural violence of poverty, environmental degradation, lack of access to health care services and unsafe working conditions - to seemingly modifiable behavioural risk factors such as tobacco use.

Patterns of population health are changing in many parts of the world, particularly in low- and middle-income countries [3,4]. In the case of Latin America, the past fifty years have been positive ones in terms of overall levels of population health, with most countries experiencing improvements in life expectancy and reductions in infant mortality rates. Yet the coming years will see increased pressures from a range of diseases that, although traditionally depicted as being diseases of affluence, actually strike along the fault lines of social inequality. If these diseases are not controlled, they will severely limit the economic development of the region [5] and cast further doubt on its ability to decrease the percentage of the population that lives in poverty. Chronic diseases will exacerbate existing inequalities [6,7].

A critical understanding of what is at stake requires a truly inter-disciplinary lens, one that integrates insights from biomedicine with epidemiological analysis and at the same time conceptualizes health issues within their historical, political, and social contexts [8]. The solutions to the health problems of the twenty-first century will require more than ever-expanding biomedical/pharmacological treatments. They will require concerted efforts aimed at the fundamental causes of disease: the social determinants of health.

The core of social science's contribution to our understanding of health and illness centers on the notion that health is produced not just by access to health care services, but by a wider set of factors rooted in the economic, political, and cultural dimensions of society [8,9]. Understanding how social determinants of health interact to ultimately shape a population's pattern of disease is a daunting task, full of methodological complexity and theoretical uncertainty. At its best, social science - including sociology, anthropology, and political science - offers us analytical tools we need to examine the complex determinants of the conditions which the World Health Organization (WHO) [4] identifies as the leading causes of death in the world: chronic, non-communicable diseases. Social science may also lead us to creative solutions to the challenges raised by these illnesses. Investigating this notion, this paper (a) outlines the global burden of chronic non-communicable diseases, highlighting their importance for population health in Latin America; (b) examines some of the common myths or half-truths surrounding these diseases; and (c) brings to the fore the notion of health inequity and why it is such an important aspect of any attempt to understand chronic non-communicable diseases.

### The Burden of Chronic Diseases

Epidemiological research indicates that chronic non-communicable diseases are the most important drivers of population health in the world [10-13]. Estimates from 2005 indicate that 35 million people died from heart disease, stroke, cancer, and other chronic conditions in that year [14,15]. Indeed, recent WHO data suggests that chronic diseases (including heart disease, stroke, cancer, respiratory diseases, and diabetes) account for 60% of the world's deaths, and that close to 80% of these deaths occur in low- and middle-income countries [4]. That is, out of the approximately 58 million deaths worldwide in 2005, 35 million were due to chronic diseases: "double the number of deaths from all infectious diseases (including HIV/AIDS, tuberculosis and malaria), maternal and perinatal conditions, and nutritional deficiencies combined" [4].

This burden is projected to increase substantially in the decades to come [16-19], severely diminishing the economic potential of low- and middle-income countries and thwarting efforts to reduce poverty. By the year 2030, the leading causes of death in the world are projected to be ischaemic heart disease, cerebrovascular disease, and chronic obstructive pulmonary disease (COPD). The leading infectious diseases - HIV/AIDS, tuberculosis, and malaria - are expected to decrease in their standing relative to chronic conditions such as diabetes mellitus and lung cancer [2]. This is not to suggest that efforts to combat infectious diseases are no longer needed. Indeed, the work of Paul Farmer [20] reminds us all of the all-too clear effects of tuberculosis and HIV/AIDS in Latin America. Treatable infectious diseases, including Chagas, continue to strike at the poor [21-23].

The characteristics of the diseases driving patterns of population health are traditionally analysed in relation to the epidemiological transition [18,24]. First developed by Omran [25-27], this model describes a change in a country's leading causes of death from infectious (or communicable) to chronic (or non-communicable) diseases. What is particularly troubling in the case of Latin America is the co-existence of significant levels of *both *types of diseases. As Banatvala and Donaldson note, "[t]he coexistence of a substantial burden of cancer, vascular disease, diabetes mellitus, and arthritis with HIV, tuberculosis, and malaria would challenge even the most mature and well-resourced health-care system" [28]. Across Latin America and the Caribbean, chronic non-communicable diseases (most notably cardiovascular diseases and cancers) account for the majority of deaths, whilst infectious diseases account for less than one-quarter of total deaths (see table 1).

**Table 1:** Distribution of total deaths (3,537,000) by major causes, Latin America and the Caribbean, 2000

Major Cause	Proportion of deaths
Communicable diseases	24%
	
Non-communicable diseases	
Cardiovascular diseases	31%
Cancers	14%
Diabetes mellitus	3%
Mental health	1%
Injuries	13%
Other non-communicable	14%

Estimated by the Global Burden of Disease Study.

*Note*: The countries included are Anguilla, Antigua and Bermuda, Argentina, Aruba, Bahamas, Barbados, Belize, Bolivia, Brazil, British Virgin Islands, Cayman Islands, Chile, Colombia, Costa Rica, Cuba, Dominica, Dominican Republic, Ecuador, El Salvador, French Guiana, Grenada, Guadalupe, Guatemala, Guyana, Haiti, Honduras, Jamaica, Martinique, Mexico, Montserrat, Netherlands Antilles, Nicaragua, Panama, Paraguay, Peru, Puerto Rico, Saint Kitts and Nevis, Saint Lucia, Saint Vincent and the Grenadines, Suriname, Trinidad and Tobago, Turks and Caicos Islands, Uruguay, US Virgin Islands, and Venezuela. *Source*: Perel et al [45].

Health care systems in Latin America struggle to meet the challenges of this wide range of disease; they face a persistent burden from infectious diseases and a growing pressure from chronic diseases. This dual burden of disease is perhaps best understood by a comparison of the years of life lost in specific countries by type of cause (see table 2).

**Table 2:** The burden of chronic diseases (selected Latin American countries)

	Distribution of years of life lost by broader causes (% of total)			Age-standardized mortality rates by cause (per 100,000 population)			
Country	Communicable diseases	Non-communicable diseases	Injuries	Non-communicable diseases	Cardio-vascular	Cancer	Injuries
Argentina	18	66	17	521	212	142	52
Bolivia	55	34	11	824	260	256	80
Brazil	30	50	20	712	341	142	81
Chile	17	64	19	453	165	137	50
Colombia	25	35	40	511	240	117	141
Ecuador	37	42	21	576	244	129	89
Paraguay	45	39	16	598	291	141	57
Peru	43	42	15	584	190	175	69
Uruguay	12	72	15	518	208	170	55
Venezuela	24	45	32	496	241	107	90
**Income group (worldwide)**							
Low income	70	20	10	754	418	114	116
Lower middle income	34	48	18	668	324	136	81
Upper middle income	30	51	19	728	436	138	102
High income	8	77	15	419	173	136	42

*Source*: WHO [2].

A close reading of the data in table 2 reveals important differences within the region. In some countries, including Argentina, Brazil, and Chile, non-communicable diseases result in the greatest number of years of life lost, in comparison to communicable diseases and injuries. In other countries, including Bolivia, Paraguay, and Peru, communicable diseases exert the more prominent influence on years of life lost. This reflects underlying patterns associated with economic development as modelled by the epidemiological transition. As shown in table 2, the balance of the burden between communicable and non-communicable diseases varies greatly by income group classification (these data reflecting the situation worldwide, not just in Latin America). In low income countries, communicable diseases exert the most important influence on years of life lost, and this balance changes quite quickly - even in lower-middle countries, non-communicable diseases amount to a heavier toll, reflecting the model of the epidemiological transition. Age-standardized mortality rates by cause also reveal important within-region differences, with cardiovascular diseases extolling a particularly heavy burden in Brazil and Venezuela.

Although per capita health expenditure on health (combined public and private expenditures) has increased between 2000 and 2005 in all country-income groups (see table 3), they fall far short of the expenditure levels in high-income countries.

**Table 3:** Health care expenditure, 2000/2005

Country	Total Expenditure on health as % of gross domestic product		General government expenditure on health as % of total experience on health		Per capita total expenditure on health (PPP int. $)	
	**2000**	**2005**	**2000**	**2005**	**2000**	**2005**

Argentina	8.9	10.2	55.4	43.9	1120	1529
Bolivia	6.1	6.9	60.1	61.6	149	203
Brazil	7.2	7.9	40.0	44.1	572	755
Chile	6.2	5.4	48.7	51.4	576	668
Colombia	7.7	7.3	80.9	84.8	485	581
Ecuador	4.2	5.3	31.2	40.0	157	274
Paraguay	9.2	7.3	40.2	36.5	336	312
Peru	4.7	4.3	53.0	49.0	228	274
Uruguay	10.5	8.1	33.4	42.5	968	885
Venezuela	6.0	4.7	53.1	45.3	356	325
						
**Income group (worldwide)**						
Low income	4.2	4.6	28.0	25.9	56	84
Lower middle income	4.6	4.8	43.4	44.9	183	295
Upper middle income	6.2	6.6	52.5	53.2	505	705
High income	10.0	11.2	59.7	60.1	2744	3712

*Source*: WHO [2].

As shown in table 3, the countries of Latin America have experienced substantially different trajectories in the recent past with respect to health care expenditures. In some countries, including Argentina, Bolivia, Brazil, and Ecuador, health care expenditure as a percentage of Gross Domestic Product has increased. In other countries, including Chile, Colombia, and Peru, it has declined (importantly, these statistics are based on different and changing denominators, and as such, health care expenditure as a percentage of Gross Domestic Product is not by itself a clear-cut signal of health expenditures). Perhaps most telling is per capita expenditure on health (see table 3); in that case, most countries in the region have experienced rising levels of expenditure.

Along with the pressure that chronic diseases bring to the health care system, they are also troubling due to their significant macro-economic effects, particularly because the available epidemiological data points to cardiovascular diseases striking younger working-age people in low- and middle-income countries [29]. For example, Abegunde et al's [5] analysis of the disease burden and loss of economic output associated with chronic diseases in 23 selected countries (including Argentina, Brazil, Colombia, and Mexico) suggests that between 2006 and 2015, chronic diseases will result in US$84 billion lost economic productivity (with approximately US$47 billion of this loss occurring in China, India, and Russia), an incredibly high burden which undoubtedly will limit the feasibility of large-scale poverty alleviation efforts. Table 4 presents the results for the Latin American countries in their study. Mexico, Brazil, Argentina, and Colombia are all expected to experience substantial reductions in potential GDP as result of the main chronic diseases in the next ten years.

**Table 4:** Projected foregone national income due to heart disease, stroke, and diabetes

	Foregone GDP (US$ billions)		Cumulative GDP loss (US$ billions) by 2015
	2006	2015	
Argentina	0.13	0.16	1.40
Brazil	0.33	0.50	4.18
Colombia	0.07	0.10	0.82
Mexico	0.48	0.89	7.14

*Note: *Adapted from Abegunde et al [5].

Building from the WHO's *Global Burden of Disease *study, and acknowledging that much uncertainty remains concerning the quantification of the comparative burden of diseases around the world, their analysis suggests that chronic diseases can be expected to result in the loss of approximately US$13.5 billion in Argentina, Brazil, Colombia, and Mexico alone (see table 4). Abegunde et al point out that "two major factors account for the grim forecasts on the economic effect of chronic diseases: the lost labour units because of deaths from chronic disease and the costs of treating chronic disease, which continue to increase annually..." [5].

Despite this burden, chronic diseases are not explicitly addressed in the Millennium Development Goals [29,30]. Yet they are a critical challenge facing the region, one that must be understood not only from the perspective of forgone national income and 'lost labour units' but also from the perspective of social justice.

### Common Myths Surrounding Chronic Diseases

Many of the WHO's advocacy efforts have been dedicated to combating commonly-accepted myths surrounding chronic diseases:

1. Chronic diseases mainly affect high income countries

2. Low- and middle-income countries need to focus their attention on infectious diseases first and chronic diseases second

3. Chronic diseases are diseases of affluence; they mainly affect rich people

4. Chronic diseases are diseases of old age

5. Chronic diseases mainly affect men

6. They are the result of individual choices - 'unhealthy lifestyles'

7. Nothing can be done to prevent them

8. Prevention and control, when possible, is too expensive [4]

In fact, the best recent data support none of these myths. As the WHO points out, four out of every five chronic disease deaths occur in low- and middle-income countries. The burden of these diseases is therefore a particular concern in the 'developing' world. Across Latin America and the Caribbean, chronic non-communicable diseases account for the majority of deaths, whilst infectious diseases account for less than one-quarter of total deaths (see table 1). The second myth - that low- and middle-income countries need to focus their attention on infectious diseases first and chronic diseases second - is based at least in part on concern for scarce resources, the argument being that in the context of limited funds, infectious diseases need to be addressed as a public health priority. However, this ignores the on-the-ground complexity; both infectious and chronic diseases shape patterns of population health and overwhelming burden of disease - as measured in proportion of total deaths - is from chronic diseases. The challenges of epidemiologic overlap were recently discussed by Waters [27] using data from Ecuador, a country near the middle of the economic and health ranking in Latin America. Waters describes epidemiologic overlap as a 'double bind', wherein infectious and communicable diseases are not completely controlled, and at the same time, opportunities to detect and treat non-communicable diseases are fragmented by socioeconomic status. However, this does not suggest that chronic conditions should be seen as a second-line priority in the region [31]. Instead, Waters' analysis of the situation in Ecuador indicates that the complexity of the epidemiologic overlap needs to be an integral component of health system planning in the region.

The third myth suggests that chronic diseases are diseases of affluence, that they mainly affect the rich. The WHO states that in all but the least developed countries of the world, chronic diseases actually run along lines of social inequality. That is, they affect the poor more than the rich, following what medical sociologists and epidemiologists refer to as the social gradient. According to Daniels et al, "...the fact is that health inequalities occur as a gradient: the poor have worse health than the near-poor, but the near-poor fare worse than the lower middle class, the lower middle class do worse than the upper middle class, and so on up the economic ladder. Addressing the social gradient in health requires action above and beyond the elimination of poverty." [32] These gradients are firmly documented in the industrialized world. Research published in recent years supports the social gradient model in Latin America - with, for example, clear gradients for both men and women in chronic disease risk factors by educational attainment in Brazil [33] and Chile [34].

The notion that chronic diseases affect mainly the elderly is also misleading: "...almost half of chronic disease deaths occur prematurely, in people under 70 years of age. One quarter of all chronic disease deaths occur in people under 60 years of age" [4]. In the case of low- and middle-income countries, the leading category of chronic disease, cardiovascular diseases, strikes particularly hard among working-age people. Tobacco use and obesity - two of the major risk factors for chronic disease - threaten the health of children and young adults [29]. According to the WHO, in low- and middle-income countries "...middle-aged adults are especially vulnerable to chronic disease. People in these countries tend to develop disease at younger ages, suffer longer - often with preventable complications - and die sooner than those in high income countries" [4]. Alongside the myth that chronic diseases are diseases of old-age, the myth that chronic diseases mainly affects men ignores the significant burden these diseases pose for women. The most recent data indicate that chronic diseases affect men and women about equally [4].

Victim-blaming, or the notion that chronic diseases are the result of individual choices in favour of unhealthy lifestyles, is particularly common. Itself a reflection of epidemiology's traditional focus on individual-level risk factors [35], this myth ignores social context; it ignores the social dimensions that underlie exposure to health-related risks that shape patterns of morbidity and mortality in all populations. For the WHO, "[t]he truth is that individual responsibility can have its full effect only where individuals have equitable access to a healthy life, and are supported to make healthy choices. Governments have a crucial role to play in improving the health and well-being of populations, and in providing special protection for vulnerable groups" [4]. Above all, this myth ignores the very real constraints placed upon individual agency by structural violence [20]. The last two myths - that nothing can be done to prevent chronic diseases, and programs for their control are too expensive for low- and middle-income countries - are particularly damaging to efforts to improve population health in Latin America, and neglect the documented effects of smoke-free legislation in the region [36,37].

### Chronic Disease and Health Inequity

In order to fight the myths surrounding chronic diseases, we need high-quality data and theoretical frameworks with which to analyze it. Above all, we need research that places utmost importance on health inequities - inequalities, or differences, that are avoidable, unnecessary, and unfair [38]. These constitute a central component of medical sociology and a growing concern in epidemiology.

Overall, a sociological perspective on population health brings our research gaze to two inter-related issues: the social determinants of health and a focus on inequity. Both issues are central to understanding the social dimensions of chronic diseases in Latin America. The social determinants of health emphasize that health is produced largely outside of the formal health care system [32]. While the formal health care system is undoubtedly crucial in improving the quality of life of people with illness, and ensuring access to health care services remains one of the pressing challenges facing all countries, the social determinants of health brings our attention to the very organization of society and the quality of social relations as a source of health (or illness).

A concern for inequities in health brings to light the social patterning of disease. Morbidity and mortality are not randomly distributed in a population; contextual factors (e.g., qualities of the places in which people live) as well as compositional characteristics (i.e., characteristics of individuals themselves) are important determinants of health. Research from this perspective attempts to overcome the limitations of a narrow individual-level analysis, but simultaneously emphasizes that recognizing the aggregate burden of chronic diseases is not enough [39,40]. This perspective says that data on the social patterning of chronic disease outcomes and risk factors are needed in order to develop effective policy responses. Such data could be used to identify regions, communities, and groups that have a high prevalence of risk factors or suffer from particularly high rates of specific disease outcomes. The 'average/deprivation/inequality' (ADI) framework - first described by United Nations Development Programme (UNDP) in its 2000 *Human Development Report *and utilized by De Maio et al in relation to chronic disease in Argentina [41] is useful in this task (see table 5).

**Table 5:** The 'average/deprivation/inequality' framework

Period	Average perspective	Deprivation perspective	Inequality perspective
One period (cross-sectional)	- What is the national average?	- Who shows the highest level of risk factors?	- What is the disparity between the least healthy and healthiest?
Over time (longitudinal)	- How has the national average changed over time?	- Has the situation of the most deprived improved over time?	- Has the difference between the least healthy and the healthiest narrowed or increased over time?

*Adapted from*: UNDP. [43]. *Human Development Report*. New York: Oxford University Press. See also De Maio et al [40].

Much of the literature on risk factor data currently falls into the "cross-sectional/average" perspective by reporting national prevalence rates. This is clearly very important, and if repeat cross-sectional or longitudinal surveys are carried out, changes in the national average could be detected. This is a crucial aspect of any attempt to evaluate relevant public policies. However, to understand the social patterning of chronic disease outcomes and risk factors, the second and third steps of the ADI framework are needed. The deprivation perspective seeks to break down the national average by relevant socioeconomic and demographic factors in order to identify the group(s) who experience either the poorest levels of health or the highest levels of risk. In other words, the deprivation perspective seeks to disaggregate national summary statistics by meaningful sociological and/or geographical levels in order to identify the segments of society experiencing the heaviest burden.

Analyses based on the ADI framework hold tremendous policy potential; they allow us to develop programs aimed to serve the worst off, and in a way, foster principles of social justice. The inequality perspective takes this one step further, not only identifying the worst-off, but also considering the *difference *between the worst-off and the best-off group. This is particularly important when it comes to public health interventions, which have an unfortunate history of sometimes increasing inequities as an unintended consequence of its actions [42]. This would enable analyses of health inequities grounded in the pursuit of social justice. It would also enable researchers to evaluate policies, model the costs/benefits of interventions, and assess the progressive realization of health as a human right.

The ADI framework was originally designed to examine the progressive realization of indicators of human rights and development. The UNDP used it to analyze inequalities by sex, education, and indigenous status in immunization rates in Egypt, literacy rates in India, and under-five mortality rates in Guatemala [43]. De Maio et al [41] applied the ADI to data on diabetes and obesity from Argentina's first National Risk Factor Survey, and demonstrated the statistical feasibility of using logistic regression results to identify the worst-off and best-off ideal types based on socioeconomic indicators and demographics. In their analysis, both income and educational attainment demonstrated statistically significant gradient-like relationships with health outcomes - suggesting that ADI-based analyses elsewhere in the region may well need to incorporate both measures. Argentina's Ministry of Health has recently carried out a follow-up survey, the 2009 National Risk Factor Survey. This opens the possibility of a longitudinal ADI analysis in that country.

At the same time, other countries in Latin America have carried out National Risk Factor Surveys, including the Southern cone countries of Brazil, Chile, Paraguay, and Uruguay, as well as Colombia, Mexico, Panama, Peru, Cuba, and some Caribbean countries. Most of these surveys have been carried out in the past 5 - 10 years, and have many questions in common, as they are based on a WHO-recommended instrument. Countries such as Chile have made differences in health outcomes between indigenous and non-indigenous peoples a priority [44]. These differences could be tracked over time using the ADI approach. There is tremendous potential for between- and within-country analyses of the socioeconomic patterning of the burden of chronic diseases in Latin America. And given the considerable heterogeneity that exists in the region - in terms of health care system design, economic policy, economic development, and ethnic composition - there is also scope for identifying 'natural experiments', or regions that deviate from expected health profiles - offering clues as to how global/regional forces interact with local context to shape public health.

Latin America's National Risk Factor Surveys offer great insight into the social patterning of chronic diseases in the region. Their value, however, will only be maximized through careful, theory-based, analysis. At the same time, these surveys can be augmented by linking to other data sources - including census data, disease-registry information, as well as other social surveys, including the World Bank's Living Standards Measurement Surveys. Many of these datasets can be harmonized to generate ecological, or area-based, indicators of socioeconomic conditions that could be incorporated in multilevel analyses.

## Conclusion

A focus on inequities would greatly advance our understanding of the burden of chronic diseases in Latin America. The aggregate-level indicators published by the WHO, disturbing as they are, take on a higher degree of urgency if we recognize that they *hide *the substantial inequities that exist in all Latin American countries.

We are faced with a unique opportunity to not only develop policies to improve aggregate-level health indicators in Latin America but also to contribute to the alleviation of the social inequality characteristic of the continent. Without significant action to address the growing burden of chronic non-communicable diseases, Latin America - and particularly the poor of Latin America - will experience growing levels of preventable morbidity and premature mortality. Research into chronic non-communicable diseases in low- and middle-income settings is just beginning - but the available evidence is unambiguous in signalling the need for urgent action.

## Competing interests

The authors declare that they have no competing interests.
